# Ammonia and Ammoniacal Salts

**Published:** 1856-08

**Authors:** 


### DIVERS FORMULÆ FOR THE EMPLOYMENT OF THE AMMONIA, AND THE AMMONIACAL SALTS.

TRANSLATED FROM THE FRENCH BY M. MORTON DOWLER, M. D., NEW ORLEANS.

As the ammoniacal preparations are more and more commanding the attention of physicians, M. le Docteur Guepin of Nantes, communicates to his confreres the following series of formulæ, as being the fruit of a long experience in practice. SOLUTION FOR COUNTERACTING DRUNKENNESS. A man is laying on the steps of your house, dead drunk; not being able either to speak or to respond to your signs. You have only to take a glass of water, and drop in it from five to fifteen drops of liquid ammonia, and cause him to swallow this glass of water, and he will be himself, arise, go away, and you are rid of the annoyance.

BLISTERING LIQUID.—℞. *Ammoniæ*, *part.* j; *Olei*, *part.* ij; M. Take a piece of wadding of the size of the blister you wish to create; remove from one side the gummed portions, moisten freely with the above liquor the side from which the gum has been removed, and apply this vesicatory to the destined part, and in five minutes the effect will be produced.

EXCITANT FOMENTATIONS FOR THE EYES.—℞. *Ammoniæ*, ʒjss; *Alcoholis Camphoratæ*, ʒijss; *Ætheris Sulphurici*, ʒjss. *M.* Let it be kept in a ground stoppered flask. If you unstop this flask and bring it near the eyes, there will be an immediate flow of tears, the agent producing an abundant secretion from the lachrymal glands.

FORMULA FOR SEDATIVE LIQUORS.—*Aquæ*, ℥jv; *Ammoniæ*, ʒijss; *Alcoholis Camphoratæ*, ʒj; *Sod. Chlor.*, ʒjss; *M.* Compresses steeped in this liquid, have a happy effect in sprains, luxations, contusions, in erysipelas, in stings of bees and wasps. A woman was one day brought into the Hotel Dieu of Nantes, suffering from phlebitis, as the consequence of a leech bite on the ankle of the right leg, which was enormously distended with erysipelatous inflammation; and our *internes* regarded death as certain, but applied the above sedative lotion. In twelve hours the danger was over.

A VALUABLE SOLUTION IN ENGORGEMENTS OF THE WOMB. *Aqua*, ʒjx; *Ammon. Hydrochlor.*, ʒjss; *m. ft. Solutio.* Take night and morning a dessert spoonful in a glass of tilly water.

MODIFICATION OF THE ABOVE TO BE EMPLOYED IN SCROFULOUS CASES.—*Aquæ*, ℥jx; *Ammon. Hydrochlor.*, ʒjss; *Potassi Iodid.*, ʒijss; *m. ft. mist.* To be taken in the same manner.

THE SAME PREPARTION COMBINED WITH DEPURATIVE SYRUPS. ℞. *Syrup. Sarzæ Compos.*, ℥xv; *Iod. Potas.*, gr. xjv; *Ammo-*

*nia Hydrochlor.*, gr. xv. *m. ft. mist.* Take a tablespoonful night and morning in some warm drink.

The hydrochlorate of ammonia is very valuable in engorgements of the breasts, used both internally and externally; internally, under the form above indicated; and externally, to be sprinkled *very lightly* over emollient cataplasms.

OINTMENTS.—The following is one of the best resolutives that can be employed in scrofulous engorgements:—℞. *Axungiæ*, ℥j; *Ammon. Hydrochlor.*, ʒj: *Plumbi Iodidi.*, gr. xv. *m. ft. ungt.* Or thus:—℞. *Axungiæ*, ℥j; *Ammon. Hydrochlor.*, ʒj; *Camphoræ*, gr. xv. *m. ft. ungt.*

The above we have often used in the form of frictions to the vertebral column; sometimes in rickets; sometimes after the application of camphor when the vertebræ are affected, and we would produce a resolutive and irritant action; sometimes in affections of the spinal marrow. The following is an eligible formula when we would obtain a strong rubefacient action:—℞. Axungiæ, ℥j; Ammon. Hydrochlor., ʒjss.; Camphoræ, gr. xv; m. ft. ungt.

ANTI-RHEUMATISMAL OINTMENT.—℞. Axungiæ, ℥j; Ammon. Carbonatis, ʒss.–ʒjss.; Calomel, ʒj; Ext. Opii, gr. xlv; Ext. Hyoscyami, ʒjss.; m. ft. ungt. Frictions made with the above on the joints affected succeed well when they are prolonged, in cases of peasants, sailors, and laboring men, and it has well succeeded with the higher class.

In syhilitic diseases, and the disorders which succeed them, the ammoniacal salts, and especially the hydrochlorate, may, with advantage take the place of the iodide of potassium, and we may, moreover always administer the two latter in combination. The following formulæ are applicable to these diseases, and to other cutaneous affections.—℞. Aquæ, oj, Sublim. Corrosiv., gr. xij; Am. Hydrochlor., ʒjss; Potas. Iodid, ʒiij; m. ft. mist. This solution has often rendered important services in various syphilitic affections in syphilitic iritis, and in syphilitic periostitis.

Anti-Syphilitic baths. ℞. Sublimat. Corrosiv., ʒijss; Am. Hydrochlor, ʒijss; M. To be used for an ordinary bath, sufficient to immerse the body, Anti-Psoraic baths. To be made of from ʒijss. to ʒiij, of the hydrochlorate, in the ordinary quantity of water.

Anti-Syphilitic Ointment.—℞. Calomelan. ʒj; Am. Hydrochlor., ℥j; Axungiæ, ℥j; m.

As a sudorific, I prefer the acetate of ammonia to any of the other ammoniacal salts.

But we close here. The examples which we have presented, will suffice to show the extent of therapeutic power which the salts possess. Our formulæ are in reality but a summary of a chapter that we might write on this important subject, and we here conclude by remarking:—That the ammoniacal salts are exceedingly active resolutive stimulants, to which we do not resort in vain. They are attended with the immense advantage, that in introducing them into the system, we are not introducing any substance foreign to constituent principles of the organism; and these salts, moreover, are readily expelled by the bowels, the kidneys, and skin.

Some of these formulæ have been long used in the Hotel Dieu of Nantes, and we would especially signalize, as having rendered great service, those which we have recommended in engorgements of the womb.—*Journal de Medecine et de Chirurgie.*

